# 919 Syrup Alleviates Postpartum Depression by Modulating the Structure and Metabolism of Gut Microbes and Affecting the Function of the Hippocampal GABA/Glutamate System

**DOI:** 10.3389/fcimb.2021.694443

**Published:** 2021-08-20

**Authors:** Xin-Yun Tian, Jing-Wei Xing, Qiao-Qi Zheng, Peng-Fei Gao

**Affiliations:** Department of Traditional Chinese Medicine, Jinshan Hospital of Fudan University, Shanghai, China

**Keywords:** postpartum depression, GABA, gut microbiome, metabonomics, Chinese herbal medicine

## Abstract

Postpartum depression (PPD) is a mental disorder that affects pregnant women around the world, with serious consequences for mothers, families, and children. Its pathogenesis remains unclear, and medications for treating PPD that can be used during lactation remain to be identified. 919 syrup (919 TJ) is a Chinese herbal medicine that has been shown to be beneficial in the treatment of postpartum depression in both clinical and experimental studies. The mechanism of action of 919 TJ is unclear. 919 syrup is ingested orally, making the potential interaction between the drug and the gut microbiome impossible to ignore. We therefore hypothesized that 919 syrup could improve the symptoms of postpartum depression by affecting the structure and function of the intestinal flora, thereby altering hippocampal metabolism. We compared changes in hippocampal metabolism, fecal metabolism, and intestinal microflora of control BALB/c mice, mice with induced untreated PPD, and mice with induced PPD treated with 919 TJ, and found that 4-aminobutyric acid (GABA) in the hippocampus corresponded with PPD behaviors. Based on changes in GABA levels, multiple key gut bacterial species (*Mucispirillum schaedleri, Bifidobacterium pseudolongum, Desulfovibrio piger, Alloprevotella tannerae, Bacteroides* sp.2.1.33B and *Prevotella* sp. CAG:755) were associated with PPD. Metabolic markers that may represent the function of the intestinal microbiota in mice with PPD were identified (Met-Arg, urocanic acid, thioetheramide-PC, L-pipecolic acid, and linoleoyl ethanolamide). The relationship between these factors is not a simple one-to-one correspondence, but more likely a network of staggered functions. We therefore believe that the composition and function of the entire intestinal flora should be emphasized in research studying the gut and PPD, rather than changes in the abundance of individual bacterial species. The introduction of this concept of “GutBalance” may help clarify the relationship between gut bacteria and systemic disease.

## Introduction

Childbirth is an exhausting process during which females undergo a series of physical and emotional changes. Postpartum depression (PPD) is a common and serious mental health problem worldwide that is associated with significant maternal suffering. Postpartum depression is a cause of personal distress that impairs a woman’s ability to function effectively in many aspects of her life. These depressive symptoms may also recur later in life (Cooper et al., 2003; Josefsson and Sydsjö, 2007). Given that postpartum women often shoulder the primary responsibility for caring for the baby, persistent depression impairs mother-infant bonding (Muzik et al., 2013; O’Higgins et al., 2013; Lefkovics et al., 2014; Dubber et al., 2015; Tsuchida et al., 2019), which leads to numerous short- and long-term negative consequences for the offspring (Tronick and Reck, 2009; Lefkovics et al., 2014; Aoyagi and Tsuchiya, 2019).

Selective serotonin reuptake inhibitors (SSRIs) are the most commonly used antidepressants for treating postpartum depression. SSRIs play an anti-depressant role by inhibiting serotonin reuptake in the presynaptic membrane of the nerve endings, thereby increasing serotonin content in the synaptic cleft and improving the neurotransmission of monoamine transmitters. Most antidepressants are excreted at low levels from the breast milk and are usually compatible with breastfeeding. Studies have shown that perinatal exposure to antidepressants may be associated with preterm delivery, weight loss, persistent pulmonary arterial hypertension, postnatal adjustment syndrome (PNAS), heart abnormalities, and autism spectrum disorder (Becker et al., 2016). It is therefore of interest to identify drugs that can relieve postpartum depression without affecting breastfeeding or the infant.

919 syrup (919 TJ) is a Chinese herbal medicine mixture. 919 TJ has been clinically found to have a positive effect on postpartum disease by improving maternal mood, reducing the symptoms of anorexia and weakness, and improving lactation. 919 TJ has also been shown to improve the appetite and increase the weight of postpartum stressed female mice (Xing et al., 2021).

The mechanism of action of traditional Chinese medicine (TCM) compounds is unclear. TCM and its decoctions are generally administered orally. The human digestive tract is full of microorganisms, and the relationship between TCM and these microorganisms cannot be ignored. A large number of studies have shown that the composition of the intestinal flora is dynamically affected by the host’s genetic background (Sekirov et al., 2010), diet (De Filippo et al., 2010), living environment (Winglee et al., 2017) and age (O’Toole and Jeffery, 2015). At the same time, many TCM extracts have had an inhibitory effect on some pathogenic microorganisms *in vitro* by inhibiting the attachment of pathogens to the intestinal epithelium, thereby reducing cell invasion and intestinal colonization. Chinese medicine is ineffective in sterile animals, which suggests that its function relies on interactions with microflora (Kato et al., 2007). In a previous work on postpartum depression by our group, we found that postpartum depression is associated with intestinal microflora disorders in both humans (Zhou et al., 2020) and rats (Ramsteijn et al., 2020).

We therefore hypothesized that 919 TJ might play a role in alleviating postpartum depression by affecting the structure and function of the gut flora. While a preliminary experiment confirmed that 919 TJ did alter the intestinal flora (Xing et al., 2021), its true mechanism of action is unknown. We created a mouse model of postpartum depression and administered 919 TJ using a multiomic approach in the hope of confirming the proposed “key bacterial species-junction metabolites-hippocampal marker” axis of postpartum depression.

## Materials and Methods

All experimental procedures were performed following approval by the Institutional Animal Care and Use Committee of Fudan University (2020-A031-01). Efforts were made to minimize suffering and minimize the number of animals used in this work.

### Animals

Naive 7-week-old male and female BALB/c mice were purchased from Shanghai Jihui Experimental Animal Feeding Co., Ltd (Shanghai, China) and reared as SPF in the Animal Laboratory Building of the Shanghai Public Health Clinic at Fudan University (Shanghai, China). We chose this strain of mice based on previous studies that showed that BALB/c females were more vulnerable to pregnancy stress and induced depression-related behaviors (Shoji and Miyakawa, 2019). Every five mice were group-housed in plastic cages (300 × 200 × 120 mm) with paper chips for bedding. Rooms were maintained at a constant temperature (23 ± 3°C) and humidity (50% ± 10%), with consistent simulated day/night cycles (12 h/day, lights on at 8:00 am). All mice were given free access to standard chow and autoclaved water *ad libitum* throughout the experiment except during the immobilization period.

### Drug Preparation

919 TJ is composed of Actinidia chinensis (20 g), Salvia miltiorrhiza (10 g), Atractylodes macrocephala (5 g), Epimedium brevicornu (4 g), Poria cocos (4 g), Radix Bupleuri (2 g), Schisandra chinensis (5 g), Alpinia katsumadai (1.5 g), Percarpium citri (2.5 g), and Pseudostellaria heterophylla (5 g). 919 TJ was produced by Shanghai Wanshicheng Pharmaceutical Co. Ltd. (Jinshan, Shanghai, China), at a concentration of 1.25 g/mL. Previous studies using different doses of 919 TJ found that a dose of 20 mL/kg resulted in optimal drug efficacy (Xing et al., 2021), and was therefore used in this work.

### Induction of Postpartum Depression (PPD) and the Administration of 919 TJ

After acclimatization for 7 days, one female was housed with two males in each cage for a continuous 4-day period until mating occurred, after which they were singly housed in plastic cages. Pregnant females (n = 30) were randomly divided into three groups of 10 mice each: controls, PPD, and 919 TJ. Twenty days after mating, all females of the three groups were checked every morning for the presence of pups, and the date of birth was designated as postpartum day 0 (PND0).

From PND2 to PND23, each mouse in the PPD and 919 TJ groups was separated from her pups and placed into a separate cage from 13:00 to 16:00 every day. Each mouse was immobilized during this period with a plastic retainer. Saline and 919 TJ (20 mL/kg body weight) were administered once a day *via* gavage to the PPD and 919 TJ groups, respectively.

### Behavioral Experiments

All behavioral tests were performed from 17:00 and 18:00. Two depression-related behavioral experiments were used to evaluate PPD severity.

The tail suspension test (TST) was performed according to the method by Dunn and Swiergiel (2005). The mouse was suspended 30 cm above a table in a visually isolated area by adhesive tape placed approximately 1 cm from the tip of her tail. Immobility time was measured for the latter 4 minutes of a 6-min testing period. When the mouse did not move and passively hung, it was considered immobile.

The forced swimming test (FST), developed by Porsolt and colleagues, was used to assess depression-related behavior. Mice were placed in a Plexiglas cylinder (200 mm height × 140 mm diameter) filled with water at approximately 21°C up to a height of 10 cm for 6 minutes. The immobility time was recorded for the latter 4 minutes. When the mouse remained floating in the water without movement, it was judged to be immobile.

### 16S rRNA Gene Sequencing

On PND2, PND9, PND16, and PND23, fecal samples were collected from each mouse at 12 a.m. and quickly frozen in liquid nitrogen. Total genome DNA was extracted using the CTAB/SDS method, and the purity and concentration of the DNA was measured. A selected V3-V4 variable region targeted for sequencing was amplified by polymerase chain reaction (PCR) using barcode-specific primers and high-fidelity DNA polymerase. PCR products were detected using electrophoresis on 2% agarose gel, and the target fragment was gel-cut and recovered using the AxyPrepDNA Gel Recovery Kit (Axygen). PCR amplification products were quantified using the Quantifluor™-ST Blue Fluorescence Quantitative System (Promega Inc.) based on the preliminary quantitative results of the electrophoresis and mixed at an appropriate proportion based on the sequencing volume requirements of each sample. Sequencing libraries were generated using the NEB Next^®^ Ultra™ DNA Library Prep Kit for Illumina (NEB, USA). Library quality was assessed with the Qubit@2.0 Fluorometer (Thermo Scientific) and the Agilent Bioanalyzer 2100 (Agilent Technologies, Santa Clara, CA, USA). Finally, the library was sequenced using an Illumina HiSeq2500, and 300bp paired-end reads were generated.

### Metagenomic Sequencing

#### Sample Testing

Two methods were used for DNA sample quality control: (1) DNA degradation degree and potential contamination was monitored with 1% agarose gels; and (2) DNA concentration was measured using the Qubit^®^ dsDNA Assay Kit and the Qubit^®^ 2.0 Flurometer (Life Technologies, CA, USA). The OD value was between 1.8 and 2.0, and DNA content above 1µg were used to construct the library.

#### Library Construction

A total of 1 µg of DNA per sample was used as input material for DNA sample preparation. Sequencing libraries were generated using the NEBNext^®^ Ultra™ DNA Library Prep Kit for Illumina (NEB, USA), and index codes were added to the attribute sequences for each sample. Briefly, the DNA sample was fragmented *via* sonication to a size of 350bp and the resulting DNA fragments were end-polished, A-tailed, and ligated with the full-length adaptor for Illumina sequencing *via* further PCR amplification. PCR products were purified (AMPure XP system) and the libraries were analyzed for size distribution with an Agilent Bioanalyzer 2100 (Agilent Technologies, Santa Clara, CA, USA) and quantified using real-time PCR.

#### Sequencing

Clustering of index-coded samples was performed using a cBot Cluster Generation System. After cluster generation the library preparations were sequenced with an Illumina HiSeq platform, and paired-end reads were generated.

### Untargeted Metabolomics

Feces were collected at 12 a.m. on PND2, PND9, PND16, and PND23 and quickly frozen in liquid nitrogen. Mice were euthanized on PND23 *via* intraperitoneal injection of 10% chloral hydrate in normal saline. The hippocampus was surgically removed from each mouse and frozen in liquid nitrogen immediately after dissection. To monitor the stability and repeatability of instrument analysis, quality control (QC) samples were prepared by pooling 10 μL of each sample and analyzing it together with the other samples. Analyses were performed using an UHPLC (1290 Infinity LC, Agilent Technologies) coupled to a quadrupole time-of-flight (AB Sciex TripleTOF 6600) at Shanghai Applied Protein Technology Co. Ltd.

For HILIC separation, samples were analyzed using a 2.1 mm × 100 mm ACQUIY UPLC BEH 1.7 µm column (Waters, Ireland). In both ESI positive and negative modes, the mobile phase contained 25 mM ammonium acetate and 25 mM ammonium hydroxide in water and acetonitrile (B). The gradient was 85% B for 1 min, linearly reduced to 65% over 11 min, changed to 40% over 0.1 min and maintained for 4 min, then increased to 85% over 0.1 min with a 5 min re-equilibration period.

### Statistical Analysis

#### Behavioral Experiments

Data analysis was performed using GraphPad Prism 8.0 (La Jolla, CA, USA). A Bonferroni post-test was used to measure between-group statistical significance of data following one-way analysis of variance (ANOVA). A p-value < 0.05 was considered statistically significant.

#### 16S rRNA Gene Sequencing

To confirm differences in the abundance of individual taxonomies between each group, Statistical Analysis of Metagenomic Profiles (STAMP v2.1.3) (Parks et al., 2014) was utilized. LEfSe was used for the quantitative analysis of biomarkers in different groups. This method was designed to analyze data in which the number of species is much higher than the number of samples, and to provide biological class explanations to establish statistical significance, biological consistency, and effect-size estimation for the predicted biomarkers. To identify differences in the microbial communities between the two groups, anosim and adonis were performed based on the Bray-Curtis dissimilarity distance matrices. Anosim analysis is a non-parametric test based on the Bray-Curtis algorithm that tests whether differences between groups are significantly greater than those within then so as to judge whether the grouping is meaningful. Adonis is also known as permutational MANOVA or nonparametric MANOVA. This method can analyze the explanatory degree of different grouping factors to sample differences, and the substitution test is used to analyze the statistical significance of this grouping.

#### Metagenomic Sequencing

Krona analysis, the exhibition of the generation situation of relative abundance, the exhibition of an abundance cluster heat map, principal component analysis (PCA) (R ade4 package, Version 2.15.3) decrease-dimension analysis was based on the abundance table of each taxonomic hierarchy. Differences between groups were tested using Anosim analysis (R vegan package, Version 2.15.3). Metastats and LEfSe analyses were used to identify different species between groups. Permutation testing between groups was used in the Metastats analysis for each taxonomy to obtain a p-value and the Benjamini and Hochberg False Discovery Rate was used to correct the p-value and acquire a q-value. LEfSe analysis was performed using LEfSe software (the default LDA score was 3).

#### Untargeted Metabolomics

After normalizing to total peak intensity, processed data were analyzed using the ropls R package, where it was subjected to multivariate data analyses including Pareto-scaled PCA and orthogonal partial least-squares discriminant analysis (OPLS-DA). Seven-fold cross-validation and response permutation testing were used to evaluate the robustness of the model. The variable importance in the projection (VIP) value of each variable in the OPLS-DA model was calculated to quantify its contribution to the classification. Metabolites with a VIP value >1 were further applied to Student’s t-test at the univariate level to measure the significance of each metabolite.

#### Correlation Analysis

Genes and metabolites with significantly different expression between groups were Z-score scaled and concatenated into one matrix. The correlation coefficient of all of the molecules in the matrix was calculated with the Pearson algorithm in R Version 3.5.1 (R-Foundation, Vienna, Austria).

### Data Availability

The metagenomic sequencing data have been deposited into the European Nucleotide Archive (ENA) in EBI under the BioProject accession code: PRJEB44528. The 16s rDNA sequencing data reported in this article are available at the NCBI SRA database with the Submission ID: PRJNA724834. Other data are available from the corresponding author upon reasonable request.

## Results

### The Effects of 919 TJ on the Depression-Related Behaviors of PPD Female Mice

**Figure 1:**
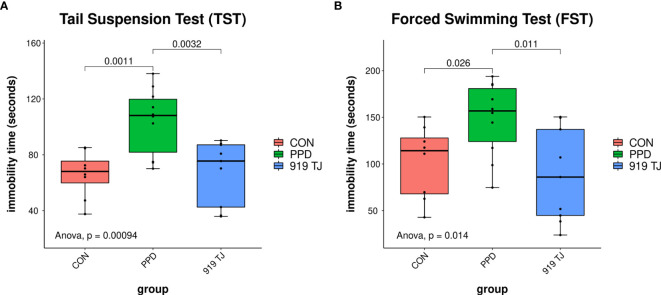
Effects of 919 TJ on the depression-related behaviors of PPD mice. The PPD model was successfully established after 3 weeks of 3-h maternal separation and restraint stress. A concurrent gavage of an equal volume of 919 TJ *vs.* saline was performed once a day. **(A)** The tail suspension test (TST) and **(B)** the forced swimming test (FST) were performed on the 23^rd^ postpartum day. Discovery set: CON, n = 9; PPD, n = 10; 919 TJ, n = 9.

Three-hours of daily separation and restraint on postpartum days 2-23 yielded a postpartum depression model with a high construct and predictive validity. Behavioral differences were observed in both the tail suspension test (TST) and the forced swimming test (FST) between the PPD group and control groups **(** **Figure 1** **)**. The longer immobility time in the PPD group compared with controls represents increased depression-related behavior. These behaviors improved in mice administered 919 TJ **(** **Figure 1** **)**.

### Changes in Hippocampal Metabolites

**Figure 2:**
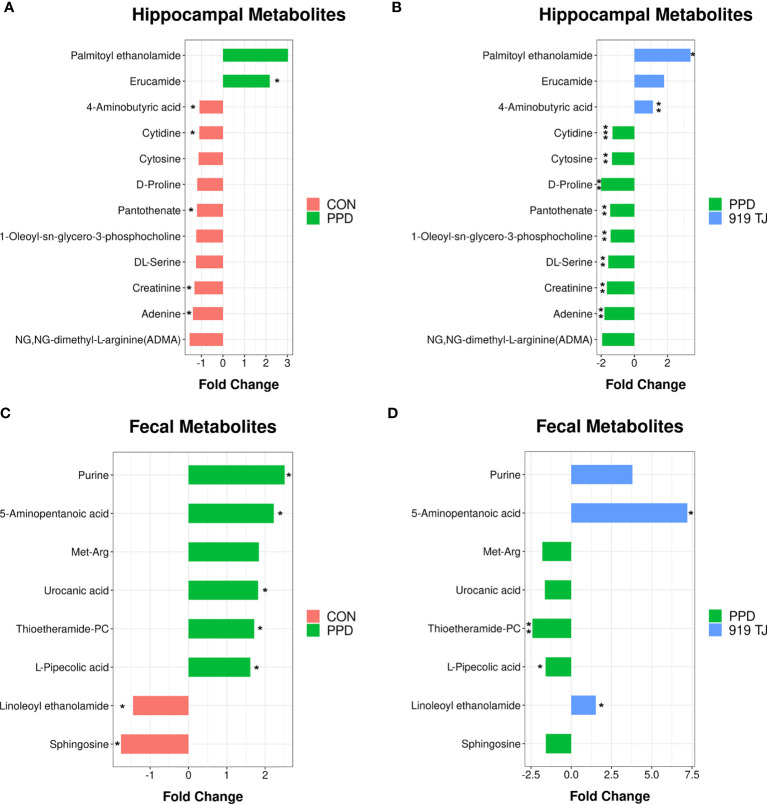
Alteration of metabolites in the hippocampus and feces. Twelve altered metabolites were selected for their differences between both the PPD group *vs.* controls **(A)** and 919 TJ *vs.* PPD **(B)**. Eight altered metabolites were selected for their variant expression in comparisons of the PPD group *vs.* controls **(C)** and 919 TJ *vs.* PPD **(D)**. Five metabolites (Met-Arg, urocanic acid, thioetheramide-PC, L-pipecolic acid and linoleoyl ethanolamide) demonstrated opposite trends in the two comparisons. Discriminative variants were identified on the basis of a VIP value > 1 and p values < 0.1. (*P < 0.05; **P < 0.01; ***P < 0.001). Discovery set: CON, n = 9; PPD, n = 10; 919 TJ, n = 9.

The PPD group displayed enrichment of 2 metabolites and depletion of 21 metabolites compared with controls, while the 919 TJ group displayed enrichment of 9 metabolites and depletion of 72 metabolites compared with the PPD group **(** **Supplementary Table 1** **)**. Eight altered metabolites were selected for comparation between inter-group differential metabolites **(** **Figures 2A, B** **)**, of which 4-Aminobutyric acid was of specific interest.

### Alteration in Fecal Metabolites

Compared with controls, the PPD group displayed enrichment of 59 metabolites and depletion of 14 metabolites. Compared with the PPD group, the 919 TJ group displayed enrichment of 20 metabolites and depletion of 19 metabolites **(** **Supplementary Table 2** **)**. According to analysis by synthesis, eight altered metabolites (purine, 5-aminopentanoic acid, Met-Arg, urocanic acid, thioetheramide-PC, L-pipecolic acid, linoleoyl ethanolamide and sphingosine) were repeated and 5 (Met-Arg, urocanic acid, thioetheramide-PC, L-pipecolic acid and linoleoyl ethanolamide) had an opposite trend in the two intergroup comparisons **(** **Figures 2C, D** **)**.

### Gut Bacteria Differences Between Groups

We obtained an average of 50,923,312 bases per sample from the whole-genome shotgun sequencing of the mouse fecal samples. Alpha-diversity was performed to evaluate the diversity of the fecal ecosystem and is a comprehensive indicator of richness and evenness. Community diversity index (including Shannon index and Simpson index) and community richness index (including Chao1 estimator and Ace estimator) were measured. There were no significant differences in these indices between the groups on the 2^nd^, 9^th^, 16^th^ or 23^rd^ postpartum day **(** **Supplementary Figure 1** **)**.

**Figure 3:**
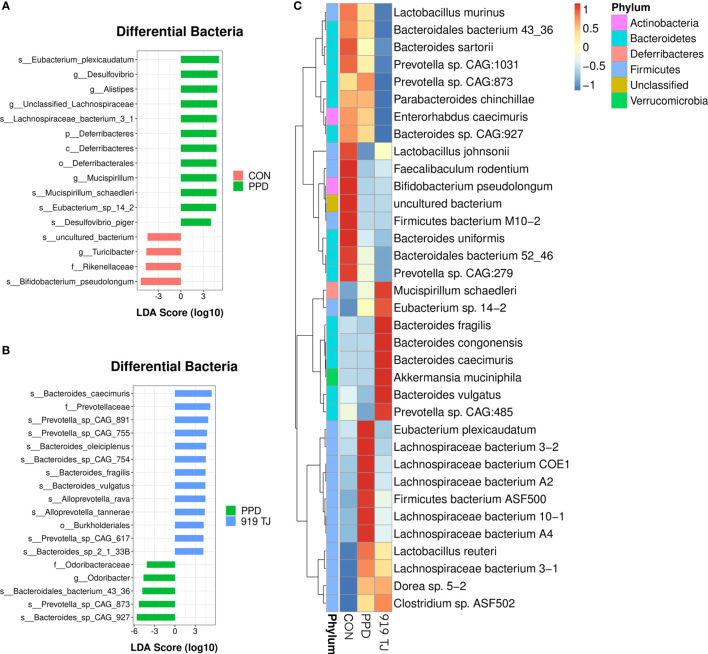
Gut bacteria differences between groups at the species level. At the species level, PPD subjects (*versus* the control group) enriched 5 species and decreased 2 **(A)**. At the species level, the 919 TJ group (*versus* the PPD group) decreased 3 bacterial species and enriched 11 **(B)**. Relative abundances of 35 bacterial species responsible for discriminating between the control, PPD, and 919 TJ groups **(C)**. Discovery set: CON, n = 9; PPD, n = 10; 919 TJ, n = 9.

We then sought to explore whether the overall bacterial phenotypes of the mice in each group were different. Anosim analysis results are shown in **Table 1-1** and Adonis results are shown in **Table 1-2**. The Anosim and Adonis analyses demonstrated that while bacterial signatures between groups were significantly different on the 23^rd^ postpartum day, no significant differences were observed on the 2^nd^, 9^th^ or 16^th^ postpartum days. PCA **(** **Supplementary Figure 2A** **)** and PCoA **(** **Supplementary Figure 2B** **)** analyses of fecal samples on the 23^rd^ postpartum day at the species level identified multiple differences in the bacterial species between the groups **(** **Figure 3C** **)**.

**Table 1-1:** Anosim analysis.

Group	R-value	p-value
CON_02 *vs* PPD_02 *vs* 919TJ_02	0.07	0.142
CON_09 *vs* PPD_09 *vs* 919TJ_09	0.079	0.114
CON_16 *vs* PPD_16 *vs* 919TJ_16	0.128	0.028*
CON_23 *vs* PPD_23 *vs* 919TJ_23	0.255	0.002**
CON_02 *vs* CON_09 *vs* CON_16 *vs* CON_23	0.018	0.327
PPD_02 *vs* PPD_09 *vs* PPD_16 *vs* PPD_23	0.172	0.003**
919TJ_02 *vs* 919TJ_09 *vs* 919TJ_16 *vs* 919TJ_23	0.095	0.043*

*P < 0.05; **P < 0.01.

**Table 1-2:** Adonis analysis.

Group	R-value	p-value
CON_02 *vs* PPD_02 *vs* 919TJ_02	0.104	0.131
CON_09 *vs* PPD_09 *vs* 919TJ_09	0.094	0.142
CON_16 *vs* PPD_16 *vs* 919TJ_16	0.11	0.054
CON_23 *vs* PPD_23 *vs* 919TJ_23	0.139	0.003**
CON_02 *vs* CON_09 *vs* CON_16 *vs* CON_23	0.088	0.445
PPD_02 *vs* PPD_09 *vs* PPD_16 *vs* PPD_23	0.141	0.007**
919TJ_02 *vs* 919TJ_09 *vs* 919TJ_16 *vs* 919TJ_23	0.118	0.051

**P < 0.01.

A total of 7 discriminative bacterial species between the control and PPD groups were identified. Compared with normal postpartum mice, PPD subjects had increased levels of 5 bacterial species (*Eubacterium plexicaudatum*, *Eubacterium* sp. 14.2, *Lachnospiraceae bacterium* 3.1, *Mucispirillum schaedleri*, *Desulfovibrio piger*) and decreased levels of *Bifidobacterium pseudolongum* and uncultured bacterium **(** **Figure 3A** **)**.

Three bacterial species declined in PPD mice treated with 919 TJ (Bacteroidales bacterium 43.36, Prevotella sp. CAG:873, Bacteroides sp. CAG:927), while 11 (Alloprevotella rava, Alloprevotella tannerae, Bacteroides caecimuris, Bacteroides oleiciplenus, Bacteroides sp. CAG:754, Bacteroides sp. 2.1.33B, Bacteroides fragilis, Bacteroides vulgatus, Prevotella sp. CAG:891, Prevotella sp. CAG:755, Prevotella sp. CAG:617) increased **(** **Figure 3B** **)**.

### Correlation Analysis Between Hippocampal GABA and Fecal Bacteria Species

**Figure 4:**
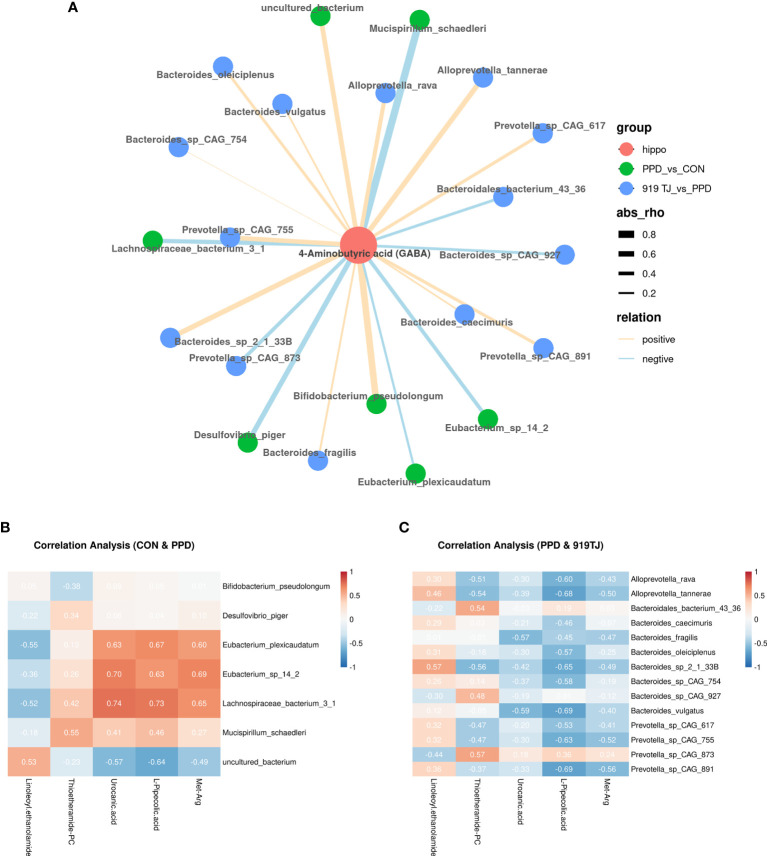
Correlation analysis between fecal bacteria species and hippocampal GABA **(A)** or fecal metabolites **(B, C)**. 4-aminobutyric acid (GABA), a key differential metabolite in the hippocampus, was anchored and a correlation analysis of the differential bacteria species was performed. The green dots represent significantly different bacterial species present in the control group *vs*. the PPD group. The blue dots represent significantly different bacterial species between the PPD group and the 919 TJ group. The magnitude of the correlation coefficient is shown by the thickness of the line. A positive correlation is in orange and a negative correlation is in blue. The heatmap reflects the degree of correlation between fecal differential metabolites (abscissa) and fecal differential bacterial species (ordinate) between CON *versus* PPD **(B)** and PPD *vs*. 919 TJ **(C)**. Red is a positive correlation, blue is a negative correlation, and the depth of the color indicates the degree of relevance. Correlation coefficients (r values) are numerically indicated.

In the PPD group, decreased *Bifidobacterium pseudolongum* and increased *Desulfovibrio piger* and *Mucispirillum schaedleri* may have the potential to reduce levels of 4-Aminobutyric acid (GABA) in the hippocampus **(** **Figure 4A** **)**.

However, 919 TJ did not change the abundance of those aforementioned species, instead increasing *Alloprevotella tannerae*, *Bacteroides* sp. 2.1.33B and *Prevotella* sp. CAG:755 to reconstruct the bacterial community and affect GABA levels **(** **Figure 4A** **)**.

### Correlation Analysis Between Fecal Metabolites and Bacteria Species

Decreased linoleoyl ethanolamide in the PPD group compared with controls may be related to the increased abundance of *Eubacterium plexicaudatum* and *Lachnospiraceae bacterium* 3.1 and the decreased abundance of other uncultured bacterium. This relationship was associated with Increased urocanic acid, L-pipecolic acid and Met-Arg. Increased thioetheramide-PC was related to the increase abundance of *Mucispirillum schaedleri* (p<0.05, 0.5<|r|≤0.8) **(** **Figure 4B** **)**.

After 919 TJ remedy, the rise in linoleoyl ethanolamide may be related to increased *Alloprevotella tannerae* and *Bacteroides* sp. 2.1.33B. The decline in thioetheramide-PC was possibly the comprehensive result of increases in *Alloprevotella rava*, *Alloprevotella tannerae*, *Bacteroides* sp. 2.1.33B, *Prevotella* sp. CAG:617, and *Prevotella* sp. CAG:755 and decreases in *Bacteroidales bacterium* 43.36, *Bacteroides* sp. CAG:927, and *Prevotella* sp. CAG:873. Decreased urocanic acid was related to increased *Bacteroides fragilis* and *Bacteroides vulgatus*. The rise in *Alloprevotella rava*, *Alloprevotella tannerae*, *Bacteroides caecimuris*, *Bacteroides oleiciplenus*, *Bacteroides* sp. 2.1.33B, *Bacteroides* sp. CAG:754, *Bacteroides vulgatus*, *Prevotella* sp. CAG:617, *Prevotella* sp. CAG:755 and *Prevotella* sp. CAG:891 together reversed the rise of L-Pipecolic acid, and the decreased level of the dipeptide Met-Arg may be associated with the increased abundance of *Alloprevotella tannerae*, *Bacteroides fragilis*, *Bacteroides* sp. 2.1.33B, *Prevotella* sp. CAG:755 and *Prevotella* sp. CAG:891 **(** **Figure 4C** **)**.

*Bifidobacterium pseudolongum* and *Desulfovibrio piger* showed an opposite trend in both quantity and function. The abundance of *Bifidobacterium pseudolongum* decreased in the PPD group, while that of *Desulfovibrio piger* increased. This may account for the up-regulation in N-acetyl-L-alanine and the down-regulation of taurine.

**Figure 5:**
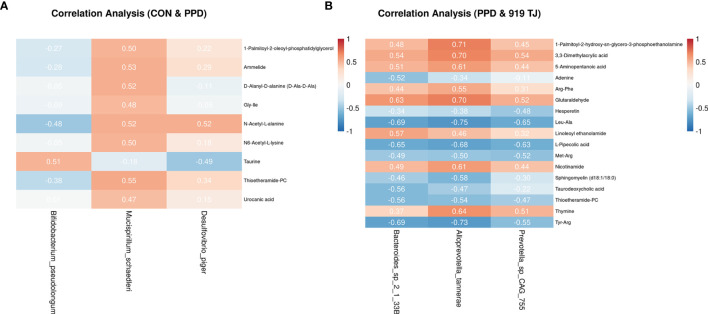
Correlation analysis between fecal metabolites and bacteria species based on GABA-associated bacteria species The heatmap reflects the degree of correlation between key bacterial species associated with GABA (abscissa) and fecal differential metabolites (ordinate) between CON *versus* PPD **(A)** and PPD *vs*. 919 TJ **(B)**. Red is a positive correlation, blue is a negative correlation, and the depth of the color indicates the degree of relevance. Correlation coefficients (r-values) are numerically indicated.

A close relationship between *Mucispirillum schaedleri* and hippocampal GABA was observed (p < 0.0001), but how these species play a key role in regulation is unclear. According to the data from the correlation analysis, *Mucispirillum schaedleri* may participate in multiple metabolic processes and its increase in PPD was possibly related to the rise in 1-Palmitoyl-2-oleoyl-phosphatidylglycerol, ammelide, D-alanyl-D-alanine (D-Ala-D-Ala), Gly-Ile, N-acetyl-L-alanine, N6-acetyl-L-lysine, taurine, thioetheramide-PC, and urocanic acid **(** **Figure 5A** **)**.

Compared with the PPD group, bacterial species relevant to GABA, including *Bacteroides* sp. 2.1.33B, *Alloprevotella tannerae* and *Prevotella* sp. CAG:755, were all increased in the 919 TJ group and took part in several metabolic processes **(** **Figure 5B** **)**.

## Discussion

The gut–brain axis has attracted increased attention as research in the field of neuroscience deepens. Thousands of publications over the past decade have reported that the trillions of bacteria in the gut could have profound effects on the brain. Based on this, we proposed that there is a keystone bacteria species, an efficient biomarker in the hippocampus, and a metabolite acting as a bridge between the peripheral and central nervous system that could be linearly connected and constitute a “bacteria-metabolite-brain” axis. However, the relationship between gut bacteria and metabolism is intricate.

In this study, a postpartum animal model using BALB/c mice was induced and depression behaviors were assessed between treatment groups and correlated with hippocampal metabolites. 4-aminobutyric acid (GABA) was then selected for focal analysis due to its potential role as a marker for assessing depression level. When we focused on bacteria species strongly associated with hippocampal GABA or postpartum depression, we identified multiple complicit metabolic processes.

### Bacteria Species Associated With the Pathogenesis of Postpartum Depression

The strong correlation between ***Mucispirillum schaedleri*** and hippocampal GABA (p<0.0001, r= -0.808) cannot be ignored. *Mucispirillum schaedleri*, the sole known member of the phylum *Deferribacteres* that is present in the gut microbiota of both mice and humans, is known to express secretion systems and effector proteins that can modify the gene expression of the host mucosa. This suggests that *Mucispirillum schaedleri* interacts intimately with the host and may play a role in inflammation (Caruso et al., 2019; Herp et al., 2019; Liu et al., 2019; Wu et al., 2020), metabolic homeostasis (Qian et al., 2020; Wu et al., 2020), and stress (Liu et al., 2019). Its clinical manifestations are disparate. *Mucispirillum schaedleri* can trigger a Crohn’s-like disease in immunocompromised hosts (Caruso et al., 2019), but was shown to be a key antagonist of Salmonella virulence (Herp et al., 2019). While it is therefore too early to define it as either harmful or beneficial, in our study the abundance of *Mucispirillum schaedleri* was negatively correlated with GABA level, potentially implicating it in the development of postpartum depression. Several differential metabolites were associated with *Mucispirillum schaedleri*, and D-Alanyl-D-alanine, N-Acetyl-L-alanine, N6-Acetyl-L-lysine, thioetheramide-PC, and ammelide all had a moderately-positive correlation with this bacteria species (p<0.05, 0.5<|r|≤0.8).

***Bifidobacterium pseudolongum*** and ***Desulfovibrio piger*** were moderately correlated with hippocampal GABA and may play an important role in the pathogenesis of postpartum depression by increasing N-Acetyl-L-alanine and reducing taurine. While the clinical implications of **N-Acetyl-L-alanine** have been identified in prior studies on primary aldosteronism (Lana et al., 2019), kidney function (Sekula et al., 2016), and rheumatoid arthritis (Takahashi et al., 2019) through metabolomic analysis, its neurobiological role has not been reported. Interestingly, **taurine** was initially thought to be an inhibitory neurotransmitter and has a close relationship with GABA. Taurine acts as a weak agonist of GABA_A_ receptors and was recently found to have different affinities to receptors composed of different GABA subunits (Ochoa-de la Paz et al., 2019). Consequently, the decline in fecal taurine may affect the level of hippocampal GABA or serve as a peripheral symbol of dysfunction in excitatory/inhibitory neurons that can be used in clinical practice. However, taurine did not increase after 919 TJ treatment.

***Bifidobacterium pseudolongum*** enhances antitumor immunity (Mager et al., 2020), exerts an anti-influenza effect (Zhang et al., 2020), enhances the efficacy of anti-CTLA4 treatment (Research, A. A for C. 2020), and treated obesity (Bo et al., 2020) in an animal model, which inspired us to explore its mechanism from the perspectives of immunity and metabolism. ***Desulfovibrio piger*** was found to be significantly increased in patients with inflammatory bowel disease (Loubinoux et al., 2002), but in recent population surveys it was used to predict preserved residual beta cell function in patients with type-1 diabetes (de Groot et al., 2021), and was found to improve hyperglycemia and reduce insulin resistance (Doumatey et al., 2020). It is therefore too early to define *Desulfovibrio piger* as a “harmful bacterium”, but it does play a negative role in the pathogenesis of postpartum depression in the present work.

### Bacteria Species Associated 919 TJ Treatment

The reduction in hippocampal GABA level caused by PPD was reversed by 919 TJ treatment, and 3 up-regulated bacterial species moderately correlated (p<0.05, 0.5<|r|≤0.8) with GABA were identified: *Bacteroides* sp. 2.1.33B, *Alloprevotella tannerae* and *Prevotella* sp. CAG:755.

The only study relevant to ***Bacteroides* sp. 2.1.33B** found that it was associated with the susceptibility genotype HLA-DRA and some other microbial markers in Vogt-Koyanagi-Harada (VKH) disease (Ye et al., 2020). Its role in neurological diseases is unknown, and therefore validation of its role in PPD is required.

***Alloprevotella tannerae***, initially isolated from the human oral cavity, was thought to be associated with dental caries (Schulze-Schweifing et al., 2014) and can be used to assess endodontic infections (Nardello et al., 2020). Considering the different localization of the bacteria in those studies compared with the present work, the role that *Alloprevotella tannerae* plays in the gut is worthy of further analysis, particularly with respect to its potential role in neurologic disease.

There has been no work to our knowledge performed on ***Prevotella* sp. CAG:755**. On the genus level, several species in *Prevotella* genus demonstrate different abundance trends **(** **Supplementary Figure 3** **)**. It therefore may not be appropriate to study them after homogenization.

Though at present there is no research that supports their role in the gut-brain axis, several potential metabolites that affected the PPD mouse were identified (p<0.05, 0.5<|r|≤0.8): Arg-Phe, Leu-Ala, Met-Arg, Tyr-Arg, thymine, nicotinamide, adenine, glutaraldehyde, hesperetin, taurodeoxycholic acid, 3,3-Dimethylacrylic acid, 5-Aminopentanoic acid, L-Pipecolic acid, thioetheramide-PC, linoleoyl ethanolamide, 1-Palmitoyl-2-hydroxy-sn-glycero-3-phosphoethanolamine, and sphingomyelin (d18:1/18:0). These metabolites provide future research directions to us as we seek to further clarify the relationship between drugs, bacteria, and disease.

### Fecal Metabolites With the Potential to Quantify the Severity of Postpartum Depression

Chinese medicine is a multi-target drug intervention, and can therefore be thought of in terms of the herbal micro-environment acting on the intestinal micro-ecology.

GABA levels are thought to be critical metabolic indicators of postpartum depression, which is consistent with the results of pre-clinical behavioral experiments. However, in clinical practice it is impractical to evaluate severity of a disease or the efficacy of a drug based on central metabolic levels. The ability for Intestinal metabolites to be significant indicators of depression is therefore worth exploring.

Urocanic acid, thioetheramide-PC, L-pipecolic acid, linoleoyl ethanolamide, and Met-Arg were thought to be worthy of additional study. Linoleoyl ethanolamide (LEA) decreased in the feces of mice with PPD and increased after 919 TJ treatment. Urocanic acid, thioetheramide-PC, L-pipecolic acid, and Met-Arg levels were positively correlated with increased levels of depression.

**Urocanic acid** (UCA), an important mediator of UV-induced immunosuppression, was found to be an inhibitor of GABA_A_ receptors (Matheson et al., 1987) and to interfere with agonist binding to GABA_A_ receptors in the brain membrane (Uusi-Oukari et al., 2000). These studies demonstrate the possibility that high levels of urocanic acid may interfere with GABA binding to receptors in the hippocampus. A recent article found that moderate UV exposure elevated blood urocanic acid, which could cross the blood-brain barrier, be converted to glutamate (GLU) and get released at the glutamatergic terminals of the hippocampus (Zhu et al., 2018). This discovery inspired us to propose that UCA is a promising pivot metabolite between intestinal microorganisms and hippocampal GABA. We therefore put forward the hypothesis that the gut microbiome is reshaped during postpartum depression. This ultimately leads to increased fecal UCA, which is delivered to the hippocampus, increases activation of the glutamate neurons and interferes with GABA binding to receptors, which manifests as an imbalance between glutamate and GABA. After intervention with 919 TJ, though no regulation of the original target was observed in either the intestinal bacteria or the hippocampal receptors and carriers **(** **Supplementary Table 3** **)**, the dysregulation of the bacterial structure and function, the imbalance between glutamate and GABA, and the animal’s depression-associated behaviors were partially reversed.

**Thioetheramide-PC** is a competitive, reversible inhibitor of secretory phospholipase A2 (sPLA2) (Yu et al., 1990). sPLA2 plays vital roles in cellular signaling and a wide number of pathophysiological situations, which range from systemic and acute inflammatory conditions to cancer (Balsinde et al., 1999, p. 2). The mRNA expression of the gene encoding for sPLA2-IIA was significantly increased in the peripheral blood cells of depressed patients *versus* controls (Gałecki et al., 2012). It was also found that the PLA2 gene increased the risk of interferon-alpha-induced depression (Su et al., 2010), and elevated PLA2 activity was observed in schizophrenics and other psychiatric patients (Noponen et al., 1993, p. 2). Interestingly, physiologic regulators of PLA2 activity: calcium, cortisol, estrogen, progesterone, and PGE2 have been found to be abnormal in patients with affective disorders, which may be similar to the paradigm of postpartum depression (Hibbeln et al., 1989). At the cellular level, exogenous PLA2 was found to provide a bidirectional modulation of the properties of the AMPA receptors (glutamate receptors) in the hippocampus (Chabot et al., 1998). PLA2 treatment of the synaptosomal membranes can inhibit the flux of chloride ions through the 4-aminobutyric acid (GABA) benzodiazepine receptor ion channel in response to activation by agonists (Koenig and Martin, 1992). Thioetheramide-PC can inhibit sPLA2 by binding to its catalytic site, and could also bind to the activator site of this enzyme. This leads to its dual effects of inhibition and activation at different concentrations (Yu et al., 1990). In our experiment, the level of fecal thioetheramide-PC was increased in the PPD group and decreased after 919 TJ. This raises several questions: (1) do elevated levels of thioetheramide-PC represent compensation for elevated central sPLA2 or represent an important link in the initiation of postpartum depression? (2) at its present concentration does thioetheramide-PC act as an inhibitor or activator? and (3) what percentage of thioetheramide-PC in the stool or blood can cross the blood-brain barrier to reach the center? While synthetic and natural inhibitors of PLA2 were found to be important for understanding and treating neurologic disorders (Ong et al., 2015, p. 2), its pathogenesis at this point still requires additional evidence.

Another metabolite worthy of attention is **L-pipecolic acid (L-PA)**. Some prior works suggested that plasma pipecolic acid does not originate from direct food intake, and can possibly be derived from intestinal bacterial metabolites (Fujita et al., 2003). L-PA was found to exert a hypnotic effect (Takagi et al., 2001) by partially activating GABA_A_ and GABA_B_ receptors (Takagi et al., 2003) by partially activating GABA_A_ and GABA_B_ receptors (Kasé et al., 1980; Nomura et al., 1981; Charles, 1986). Pipecolic acid is thought to be a neurotransmitter or neuromodulator (Chang et al., 1981; Chang and Myslinski, 1985) that plays a role in the inhibitory GABA system. According to our hypothesis, depressive behaviors are associated with reduced activation of the GABA system, so L-PA should be decreased in the PPD group. However, the results of the present work showed that L-PA increased in PPD mice and was corrected after 919 TJ. Whether this phenomenon represents compensation in the stress state or fecal L-PA reflects dysfunctional central metabolism is elusive.

**Linoleoyl ethanolamide (LEA)** was reduced in the PPD group and reversed after treatment. It was associated with mediating the acute suppression of food intake (Ho et al., 2020), as well as increased reward value of food *via* activation of PPARα in enterocytes followed by activation of afferent vagal fibers leading to the brain (Hansen, 2014). According to the results of our previous experiments (Xing et al., 2021), loss of appetite was observed in mice with postpartum depression, which seems to contradict this phenomenon. This is of particular interest to us and will be the subject of future works.

The role that **Met-Arg** plays in postpartum depression is unclear, but several attempts have been made to use dipeptides as antidepressants (Hatanaka et al., 2012; Ano et al., 2019; Mizushige et al., 2020; Nogimura et al., 2020; Gudasheva et al., 2021). A deeper understanding of dipeptides may be an important link to unravelling the relationship between gut flora and postpartum depression.

**In general**, this study sought to explore the interrelationships between gut flora, gut metabolites, hippocampal metabolism and depressive phenotypes, and joint analysis and correlation studies were performed based on multiomics. Some biomarkers and microbial species with the potential for clinical utility as markers of postpartum depression were identified. The level of 4-aminobutyric acid (GABA) in the hippocampus was introduced as a potential modality for assessing the severity of depression, and the subsequent screening of differential bacterial species was based on this. *Mucispirillum schaedleri*, *Bifidobacterium pseudolongum*, and *Desulfovibrio piger* were identified following comparisons between the postpartum depression and control groups as possible participants in the incidence of PPD. Their association with GABA may be mediated by metabolites as discussed above. *Bacteroides* sp. 2.1.33B, *Alloprevotella tannerae*, and *Prevotella* sp. CAG:755 were involved in the structural remodeling and functional restoration of the bacterial community after 919 TJ medication.

That inspired us not to stick to *in-situ* adjustment but to focus on restoration of the overall function of the intestinal microecology, which coincides with the concept of “GutBalance” (Yang et al., 2021). GutBalance posits that the quantitative composition of the microbiome is important but often neglected in abundance-based statistical analysis because it is too hard to handle. The concept of balance-disease associations rather than conventional microbe-disease associations should be highlighted. Our experiment also reflected this problem. On the one hand, PPD mice showed improved depressive behaviors after 919 TJ treatment, clinical effects that were reflected by changes in select metabolites in the hippocampus and feces. However, the bacterial species that had previously changed in the PPD group were not modulated, while the abundance of other bacterial species changed. We therefore believe that phenotypic-related intestinal metabolites are critical, and that urocanic acid, thioetheramide-PC, L-pipecolic acid, linoleoyl ethanolamide, and Met-Arg are potential intestinal markers for assessing the severity of depression. These metabolites may also be involved in regulating the onset and remission of postpartum depression. Additional replicative and causal studies are required to establish the role of intestinal microorganisms in the pathogenesis of postpartum depression.

## Data Availability Statement

The datasets presented in this study can be found in online repositories. The metagenomic sequencing data are deposited in the European Nucleotide Archive (ENA) in EBI, accession number: PRJEB44528. The 16s rDNA sequencing data are deposited in the NCBI SRA database, accession number: PRJNA724834. Other data are available from the corresponding author upon reasonable request.

## Ethics Statement

The animal study was reviewed and approved by the Institutional Animal Care and Use Committee of Fudan University (2020-A031-01).

## Author Contributions

Conception and design of study: X-YT, J-WX, and P-FG. Acquisition of data: X-YT, J-WX, and Q-QZ. Analysis and/or interpretation of data: X-YT, J-WX, and Q-QZ. Drafting the manuscript: X-YT. Revising the manuscript critically for important intellectual content: J-WX, Q-QZ, and P-FG. Approval of the version of the manuscript to be published: X-YT, J-WX, Q-QZ, and P-FG. All authors contributed to the article and approved the submitted version.

## Funding

The study was supported by the National Natural Science Foundation of China (No. 81973785).

## Conflict of Interest

The authors declare that the research was conducted in the absence of any commercial or financial relationships that could be construed as a potential conflict of interest.

## Publisher’s Note

All claims expressed in this article are solely those of the authors and do not necessarily represent those of their affiliated organizations, or those of the publisher, the editors and the reviewers. Any product that may be evaluated in this article, or claim that may be made by its manufacturer, is not guaranteed or endorsed by the publisher.

## Supplementary Material

Supplementary Figure 1 Gut bacteria differences in alpha-diversity between groups. No significant differences in alpha-diversity were observed between the control, PPD and 919 TJ groups on the 2^nd^, 9^th^, 16^th^ or 23^rd^ postpartum days. Community diversity (Shannon index and Simpson index) and community richness (Chao1 estimator and Ace estimator) indices were measured. Discovery set: CON_2, n = 9; PPD_2, n = 9; 919 TJ_2, n = 8; CON_9, n = 9; PPD_9, n = 9; 919 TJ_9, n = 9; CON_16, n = 9; PPD_16, n = 10; 919 TJ_16, n = 9; CON_23, n = 9; PPD_23, n = 10; 919 TJ_23, n = 9.

Supplementary Figure 2 Gut bacteria differences between groups at the species level. On the 23^rd^ postpartum day, bacterial signatures between groups were significantly different.

Supplementary Figure 3 The abundance of species Prevotella spp. in the 919 TJ group vs. PPD.
