# Supplementary figures and images for: 919 Syrup Alleviates Postpartum Depression by Modulating the Structure and Metabolism of Gut Microbes and Affecting the Function of the Hippocampal GABA/Glutamate System
Source: Front Cell Infect Microbiol. 2021 Aug 20;11:694443. doi: 10.3389/fcimb.2021.694443 (PMC8417790; doi:10.3389/fcimb.2021.694443)

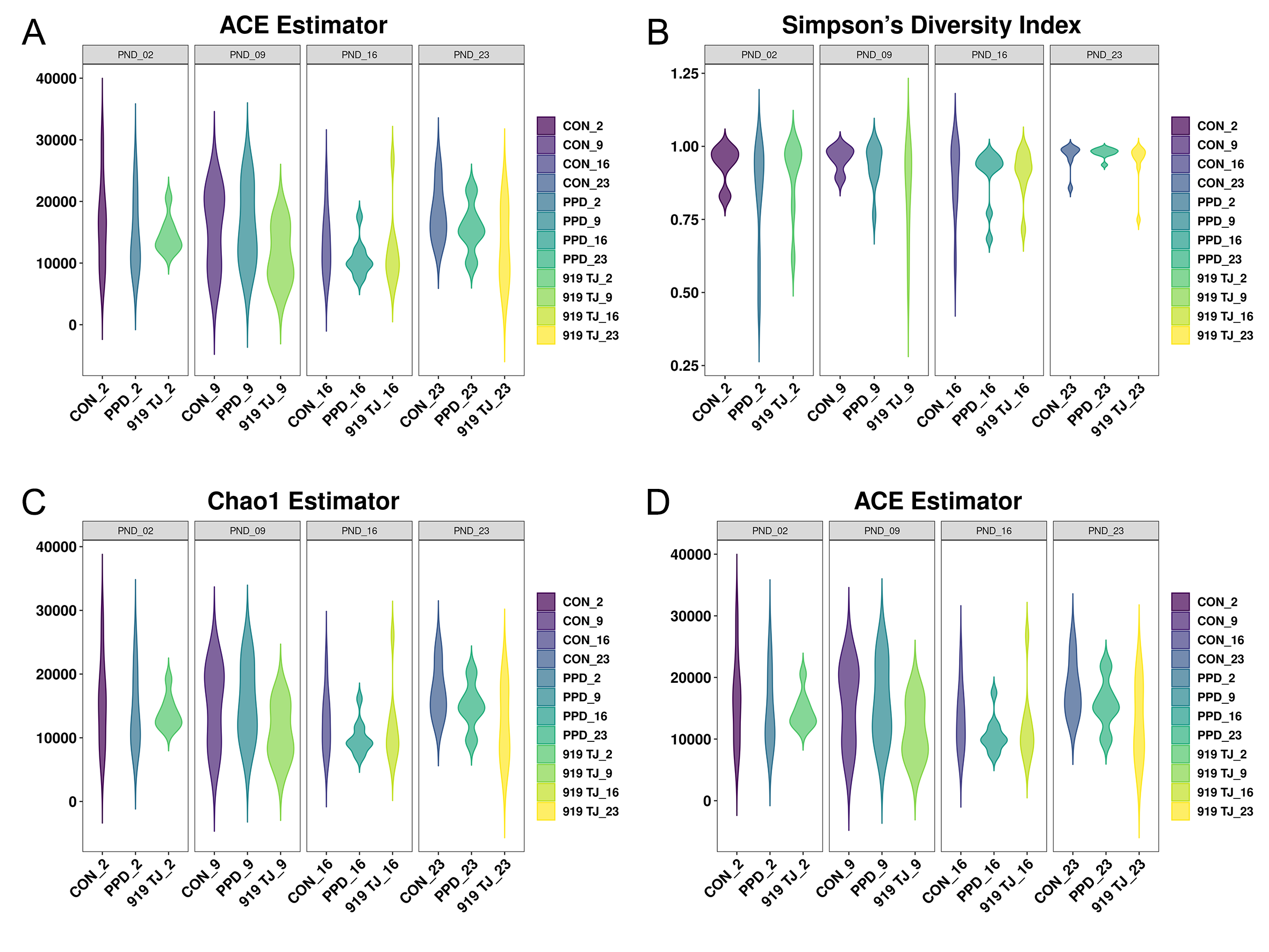

Supplement: Supplementary Figure 1 — Gut bacteria differences in alpha-diversity between groups. No significant differences in alpha-diversity were observed between the control, PPD and 919 TJ groups on the 2nd, 9th, 16th or 23rd postpartum days. Community diversity (Shannon index and Simpson index) and community richness (Chao1 estimator and Ace estimator) indices were measured. Discovery set: CON_2, n = 9; PPD_2, n = 9; 919 TJ_2, n = 8; CON_9, n = 9; PPD_9, n = 9; 919 TJ_9, n = 9; CON_16, n = 9; PPD_16, n = 10; 919 TJ_16, n = 9; CON_23, n = 9; PPD_23, n = 10; 919 TJ_23, n = 9. [file Image_1.tif]

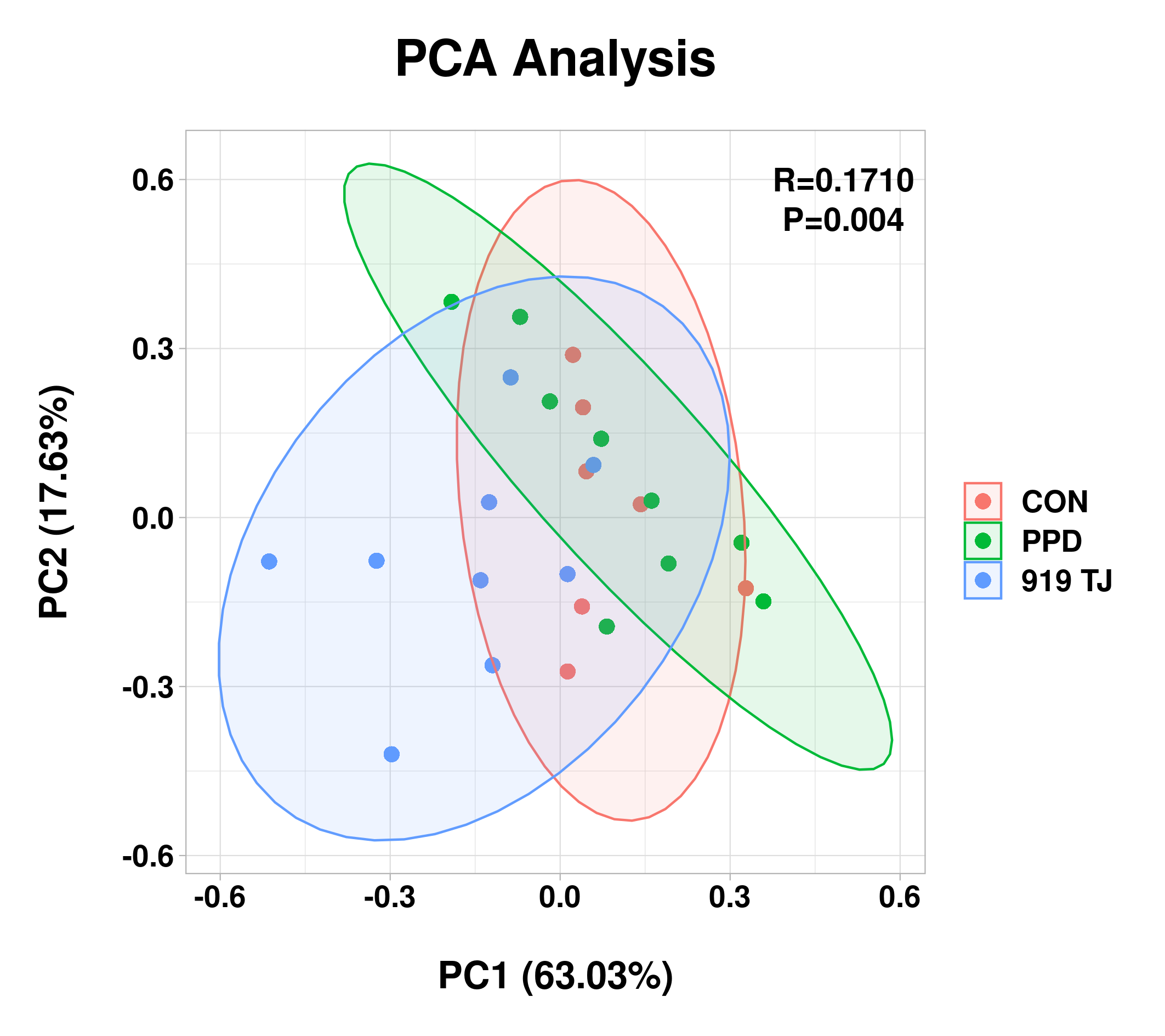

Supplement: Supplementary Figure 2 — Gut bacteria differences between groups at the species level. On the 23rd postpartum day, bacterial signatures between groups were significantly different. [file Image_2.tiff]

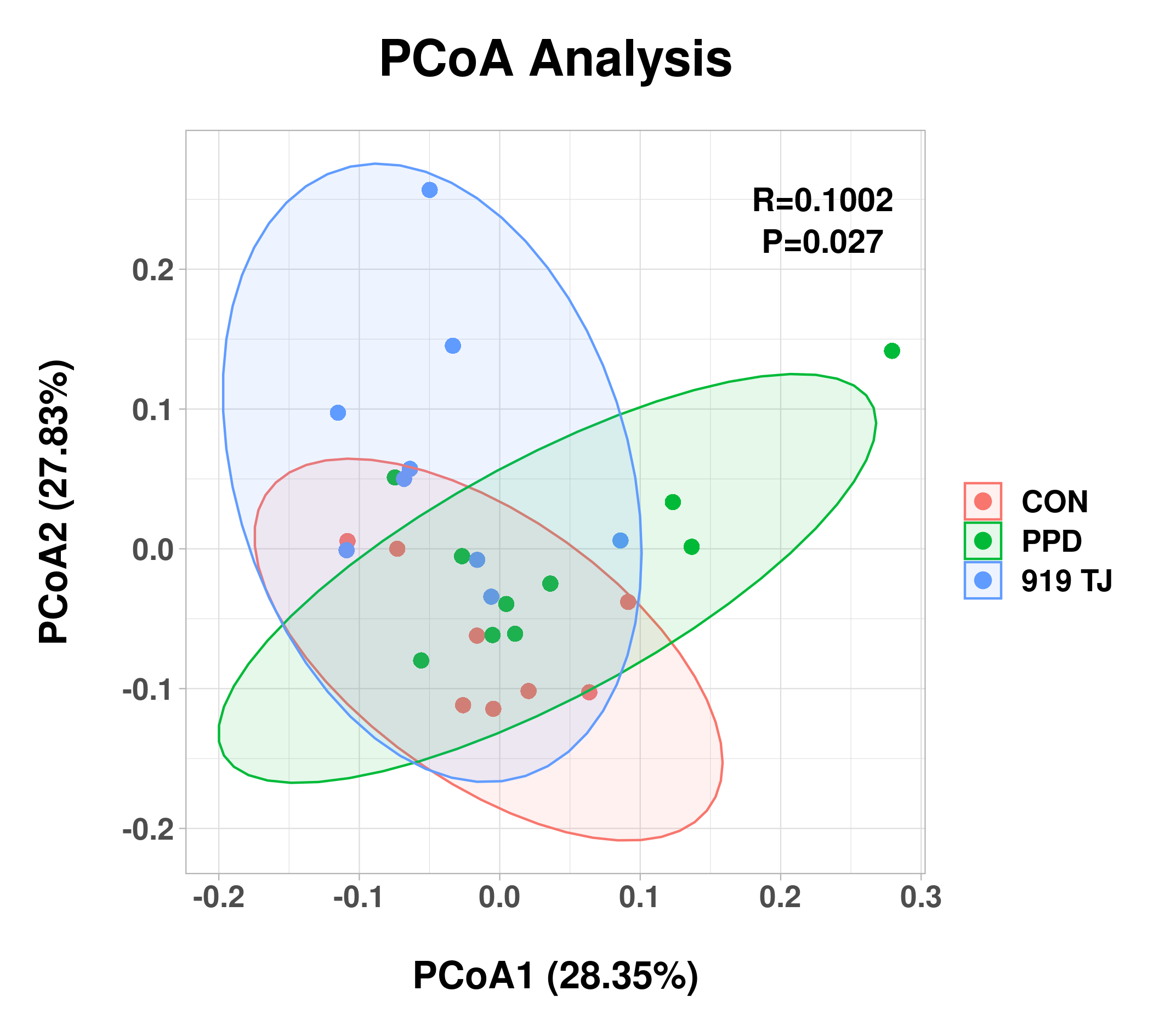

Supplement: Supplementary Figure 3 — The abundance of species Prevotella spp. in the 919 TJ group vs. PPD. [file Image_3.tiff]

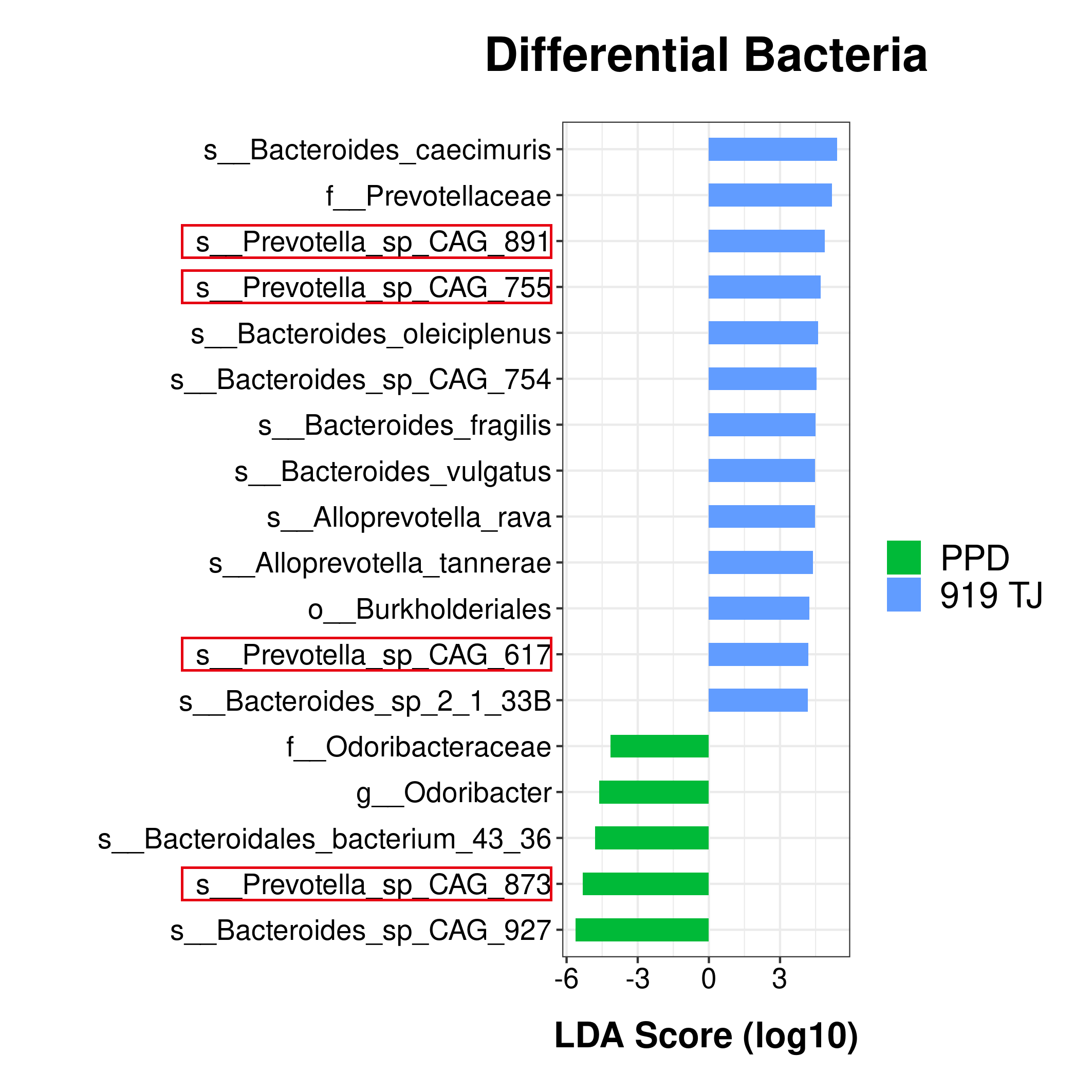

Supplement: Supplementary file 4 [file Image_4.tif]
