# Supplementary material for: 919 Syrup Alleviates Postpartum Depression by Modulating the Structure and Metabolism of Gut Microbes and Affecting the Function of the Hippocampal GABA/Glutamate System
Source: Front Cell Infect Microbiol. 2021 Aug 20;11:694443. doi: 10.3389/fcimb.2021.694443 (PMC8417790; doi:10.3389/fcimb.2021.694443)
Supplement: Supplementary file 5 [file DataSheet_1.pdf]

PPD vs CON

Name	VIP	Fold change	p-value
**NEG**			
Stearic acid	1.426060766	0.631658946	0.012593888
Pantothenate	2.279027276	0.829997602	0.021757441
3-Guanidinopropanoate	1.09219485	0.731970363	0.038926564
Cytidine	1.259100437	0.908910631	0.045592971
Uric acid	1.002318037	0.491492841	0.052325992
Lumichrome	1.119121271	0.875129503	0.052669164
cis-9-Palmitoleic acid	1.019019248	0.672323771	0.062431292
DL-Serine	1.176008849	0.799975733	0.093143269
N-Acetylneuraminic acid	1.237149067	0.779926063	0.096381633
**POS**			
15-Deoxy-delta-12,14-PGJ2	1.432395147	0.554359592	0.007454745
4-Aminobutyric acid	2.132700133	0.917297038	0.012007822
Prostaglandin I2	1.159380263	0.535455547	0.012522631
Adenine	2.570211922	0.714226241	0.01459912
4-Guanidinobutyric acid	1.225138656	0.828518646	0.032736187
Erucamide	3.481693568	2.17754978	0.036992223
Creatinine	3.502315456	0.753432045	0.041856488
Adenosine	2.741816032	0.74140593	0.052854535
Cytosine	1.970909007	0.879593031	0.058767991
2-Methylbutyroylcarnitine	1.399379777	0.729493464	0.063472615
D-Proline	3.277497358	0.836558786	0.065818469
1-Oleoyl-sn-glycero-3-phosphocholine	7.027568386	0.80575656	0.077993449
Palmitoyl ethanolamide	1.730113615	3.026801351	0.087687685
NG,NG-dimethyl-L-arginine(ADMA)	1.768134745	0.647013684	0.098756476

919 TJ vs PPD

Name	VIP	Fold change	p-value
**NEG**			
11-Keto-.beta.-boswellic acid	2.41913506	3.856281563	0.000129026
Cytidine	1.147728418	0.758586069	0.000245461
Dehydroascorbic acid (Oxidized vitamin C)	3.601492285	0.212110133	0.000317474
Taurochenodeoxycholate	3.794236022	5.307300129	0.000608271
Uracil	3.947743402	0.506219294	0.000676145
Allantoin	1.645936786	0.35765623	0.000794707
Confertifoline	3.028182677	0.44332436	0.000871981
Heptadecanoic acid	1.558687464	0.368927803	0.000964779
Linoleic acid	5.61386543	0.255035445	0.000974793
6-Keto-PGF1a	2.769420086	0.009824753	0.001047548
9R,10S-EpOME	2.933850143	0.055995875	0.001238328
DL-Serine	1.15936773	0.634140182	0.00133206
1-Palmitoyl-2-oleoyl-phosphatidylglycerol	7.249964556	0.330576391	0.001405167
D-Proline	1.426825714	0.498881806	0.00157617
L-Tyrosine	1.175385528	0.46919998	0.001670777
13-OxoODE	2.012042169	0.101470872	0.001698513
DL-Methionine sulfoxide	1.104978133	0.121420259	0.001715288
2-Oxoadipic acid	7.383584906	0.454327246	0.001786502
PGF2a	1.784634825	0.457129076	0.001904494
(+-)12-HETE	4.794180521	0.170780752	0.002333886
Adenine	1.21859038	0.554463387	0.002452287
L-Phenylalanine	3.307738275	0.508984328	0.002583331
all cis-(6,9,12)-Linolenic acid	1.252530339	0.413419773	0.002702619
Xanthosine	1.162551794	0.453592922	0.00308015
16-Hydroxypalmitic acid	1.274266258	0.302306448	0.003165999
Adenosine 3'-monophosphate	1.328472842	0.172820905	0.00352435
D-Mannose 1-phosphate	1.247591833	0.528209342	0.003703724
Acamprosate	1.542487723	0.426263433	0.005103227
L-Malic acid	2.371288218	0.600125404	0.005456301
Pantothenate	1.989544471	0.758723368	0.005854875
Norethindrone Acetate	3.511095842	0.38019483	0.007287003
Indoxyl sulfate	1.506908558	0.525368411	0.009937265
PGA1	1.172211392	0.280230058	0.010239302
Taurine	5.757514013	0.870017222	0.016430339
Embelin	2.503799801	0.528955761	0.016907985
Nname,cis-9,10-Epoxystearic acid	1.196935748	0.513019501	0.01985865
Myristic acid	1.247792407	2.727200726	0.021478653
Dihydroxyacetone phosphate	1.102719449	0.593521888	0.027427259
Taurocholate	3.446310164	2.589837894	0.02745577
11(Z),14(Z)-Eicosadienoic Acid	1.298755155	0.629490435	0.03653528
gamma-L-Glutamyl-L-glutamic acid	1.665827954	0.780599761	0.052282424
Xanthine	2.128875394	0.787026783	0.052929152
1-Palmitoyl-2-hydroxy-sn-glycero-3-phosphoethanolamine	1.770575524	0.783969813	0.066953727
D-Erythrose 4-phosphate	1.787112137	0.661257794	0.070428983
Guanosine	1.421556198	0.845113819	0.070748367
**POS**			
L-Phenylalanine	2.201254871	0.432150846	0.00020419
Tyramine	3.109367488	0.413925474	0.000331565
L-Leucine	1.7525642	0.470313332	0.000464209
D-Proline	2.419437529	0.692709665	0.000507867
Thioetheramide-PC	4.425800337	0.45487969	0.000634837
L-Carnitine	7.136373864	0.717673047	0.00074527

gamma-L-Glutamyl-L-glutamic acid	4.131063662	0.803119775	0.000769359
7-Oxocholesterol	1.701307699	0.154999014	0.000945695
Cytosine	1.597923221	0.738657325	0.00107019
1-O-(cis-9-Octadecenyl)-2-O-acetyl-sn-glycero-3-phosphocholine	1.119176096	0.671302247	0.001285903
Acetylcarnitine	5.006047425	0.354751319	0.001849062
Pantothenate	1.336903189	0.685182226	0.001849247
(3-Carboxypropyl)trimethylammonium cation	1.108894757	1.869658887	0.001936882
Cytidine	1.069920666	0.761448095	0.00202246
4-Aminobutyric acid	1.284994549	1.11091366	0.002794768
Adenine	1.626702642	0.608383079	0.00311149
Stearidonic Acid	2.839539397	0.112896447	0.00317905
DL-Indole-3-lactic acid	1.208532372	0.489288705	0.003244784
alpha-Linolenic acid	2.17168797	0.203892753	0.003583879
Cyclohexylamine	2.977119754	0.544385409	0.003597927
Palmitoyl ethanolamide	1.70251087	3.387389527	0.003910085
1-Stearoyl-2-oleoyl-sn-glycerol 3-phosphocholine (SOPC)	3.846082064	0.278163922	0.00392863
Jasmine lactone	1.285080703	0.014910807	0.00409216
Creatinine	2.477294529	0.603692281	0.00416167
1-Oleoyl-sn-glycero-3-phosphocholine	5.260784882	0.69901364	0.004340888
PC(16:0/16:0)	9.899464207	0.354388625	0.004912534
L-Palmitoylcarnitine	2.569332214	0.615346791	0.005124434
trans-Vaccenic acid	1.816596457	0.186947691	0.005519412
all cis-(6,9,12)-Linolenic acid	1.041428977	0.628401777	0.006452202
Phosphorylcholine	9.062315954	0.883220665	0.006761981
Glutathione disulfide	1.04002631	0.533403001	0.00700755
1-Palmitoyl-sn-glycero-3-phosphocholine	11.46175758	0.424441283	0.009408794
1-Methylnicotinamide	1.049229538	0.40027522	0.009806572
Guanosine	1.046937965	0.793426162	0.014298159
1-Palmitoyl-2-hydroxy-sn-glycero-3-phosphoethanolamine	1.240284581	0.790106158	0.014345164
Allopurinol riboside	1.928651756	0.841618181	0.014528817
L-Arginine	6.14606646	0.530466345	0.018605868
1-Stearoyl-2-arachidonoyl-sn-glycerol	2.466283519	0.638580665	0.020781958
Lys-Leu	1.130898389	0.883735233	0.021977706
Sphingomyelin (d18:1/18:0)	3.46480795	0.272997348	0.022223716
S-Methyl-5'-thioadenosine	1.092791856	1.373794847	0.029557289
N6,N6,N6-Trimethyl-L-lysine	1.821380434	0.755234442	0.043119349
Erucamide	2.115433272	1.795077124	0.050770104
NG,NG-dimethyl-L-arginine(ADMA)	1.117826111	0.517265455	0.060033184
1-Stearoyl-2-hydroxy-sn-glycero-3-phosphocholine	2.259529365	0.765765105	0.078025448
