# Supplementary material for: 919 Syrup Alleviates Postpartum Depression by Modulating the Structure and Metabolism of Gut Microbes and Affecting the Function of the Hippocampal GABA/Glutamate System
Source: Front Cell Infect Microbiol. 2021 Aug 20;11:694443. doi: 10.3389/fcimb.2021.694443 (PMC8417790; doi:10.3389/fcimb.2021.694443)
Supplement: Supplementary file 6 [file DataSheet_2.pdf]

PPD vs CON

Name	VIP	Fold change	p-value
**NEG**			
Manumycin A	3.912923158	0.446367078	0.002954811
N-Acetyl-L-alanine	1.066031806	2.053655394	0.00746468
N6-Acetyl-L-lysine	1.031806025	1.52612844	0.009837893
Glycyl-L-leucine	3.310142642	2.201057501	0.00996063
Sunitinib	3.21857826	0.421149984	0.015998746
L-Ascorbic acid	1.654669122	3.163568185	0.031103712
N-Acetyl-L-glutamate	2.51202814	1.425064586	0.03248841
Bisindolylmaleimide I	2.91898793	0.442766421	0.033337341
Urocanic acid	1.467251831	1.815320094	0.035298178
Sucrose	1.032289204	2.02397387	0.035967165
D-Alanyl-D-alanine (D-Ala-D-Ala)	1.467483854	2.117968385	0.036722616
Acetyl-DL-Leucine	1.238363831	1.896940144	0.037861401
1-Palmitoyl-2-oleoyl-phosphatidylglycerol	1.530886028	5.418558526	0.045095741
Ammelide	2.165501623	2.016356169	0.049617672
Adynerin	2.484280975	0.650300072	0.057270039
Hypoxanthine	5.840531621	1.5864618	0.06153573
Taurine	4.881004343	0.386255899	0.066125343
gamma-L-Glutamyl-L-phenylalanine	1.333220887	2.109905877	0.067895878
Acamprosate	7.46680645	0.405827573	0.081626153
Nicotinate	1.531277981	1.430104656	0.083878176
L-Glutamate	3.665136202	2.200396369	0.084893211
Pantothenate	5.618222122	1.670893418	0.094211053
all cis-(6,9,12)-Linolenic acid	3.332015616	0.73550605	0.095046475
ponasterone A	1.025528247	0.591331642	0.095070214
**POS**			
Phe-Gly	1.450548012	1.779400211	0.010363578
Gly-Ile	1.790496625	1.930888009	0.010507872
Ile-Lys	3.195051658	2.250172299	0.020499583
Val-Lys	2.546087972	2.128296623	0.021137922
Arg-Ala	2.800270064	1.64499038	0.023098168
Dihydrotachysterol	1.367365178	0.676851231	0.023716057
Urocanic acid	3.181598449	1.791432906	0.024081879
Ergocalciferol (Vitamin D2)	1.675835992	0.505294416	0.02452192
N-Acetyl-D-glucosamine	1.560993349	1.535023743	0.02751935
Val-Met	1.57742065	2.124937816	0.02754469
Ile-Ala	1.376710666	2.157766524	0.030709739
Met-Lys	1.110533378	1.825271514	0.031296751
Lys-Ser	1.270155769	1.927603969	0.033189779
Taurine	5.618724591	0.298859014	0.033854127
Ile-Pro	2.663441489	1.805411003	0.034822654
Ala-Leu	2.327558364	2.097348392	0.035589891
1-Palmitoylglycerol	1.839762875	1.615636601	0.037281784
Linoleoyl ethanolamide	3.954465504	0.691088049	0.038215147
Val-Pro	1.208083543	1.678715993	0.039465516
Purine	1.249880443	2.510784526	0.039672986
His-Ile	1.644078579	1.876473767	0.040664054
Ser-Val	1.041346998	1.899186224	0.040805233
Thioetheramide-PC	4.92243521	1.7162596	0.040843447
Sphingosine	7.995601209	0.568254983	0.042945395
Ile-Arg	4.02547902	1.718280697	0.044230974
Gly-Arg	1.727368211	1.582447792	0.044433034
Val-Tyr	1.58093938	1.984350863	0.044763673
Val-Val	2.265828346	3.587562877	0.046446198
L-Pipecolic acid	2.634850975	1.612068115	0.04850425
Val-Ala	1.598625217	2.186084614	0.051342595

Tyr-Ala	1.355934242	1.487917689	0.052020406
5-Aminopentanoic acid	2.768621811	2.225650552	0.053223441
Leu-Ser	1.585470179	1.770693303	0.053284677
Glycerol 1-myristate	2.959503786	1.617494015	0.054375494
Quinaldic acid	1.262085283	1.532207313	0.055556558
Phe-Phe	2.208518872	1.855937138	0.058189348
Val-Phe	2.471318726	2.012418318	0.060829381
Oxyquinoline	2.342727816	1.502853126	0.062668973
Met-Arg	1.163803394	1.836943731	0.063916141
Ile-Ile	4.885712034	2.67243699	0.06414066
Hypoxanthine	13.66287624	1.584881633	0.064689315
Val-Arg	2.712333078	1.738224744	0.064752908
L-Threonine	1.335420837	1.405149128	0.071564083
Deoxyadenosine	13.74047354	0.594692383	0.072842376
Riboflavin	1.302745842	1.643207819	0.074167128
Val-Ile	1.121102481	1.869880866	0.075772136
Thr-Arg	1.5940399	1.560740074	0.080257843
Ser-Lys	1.410022266	1.522407889	0.080914871
L-Glutamate	3.146615483	2.01325321	0.08331671
Daidzein	1.193227722	1.330476677	0.083502093
Pantothenate	2.424932575	1.650808764	0.090895958
L-Citrulline	1.858098153	1.419313448	0.092045322
Sphinganine	4.362587708	0.578392441	0.096489828
Phe-Ile	3.20810964	1.720202227	0.098056982

919 TJ vs PPD

Name	VIP	Fold change	p-value
**NEG**			
Thymine	4.109011787	1.779434495	0.015277269
Genistein	1.580804997	2.247744826	0.015403256
5-Aminopentanoic acid	5.660401036	7.206502435	0.016050952
Adenine	3.926976868	0.383947115	0.026833762
1-Palmitoyl-2-hydroxy-sn-glycero-3-phosphoethanolamine	4.940514688	1.712573343	0.030450012
Pentadecanoic Acid	6.077372455	1.708501218	0.030635813
Hesperetin	3.732560732	0.091560966	0.034927003
Maltose	1.136502358	1.710725402	0.046366558
Deoxyinosine	7.427912389	1.876220548	0.063824355
D-Quinovose	1.219589169	1.786058497	0.066974776
Taurochenodeoxycholate	5.908084466	0.272123513	0.07530651
Homoveratric acid	2.36247254	1.556304875	0.080333396
Chenodeoxycholate	2.456453057	0.628558644	0.080692557
Urocanic acid	1.179833171	0.609154721	0.098903832
**POS**			
Tyr-Arg	1.249006602	0.465878545	0.00167507
Leu-Ala	2.327747658	0.452561136	0.002236114
Thioetheramide-PC	4.214176136	0.415000331	0.004488005
Glutaraldehyde	1.360331872	3.834624849	0.007377913
3,3-Dimethylacrylic acid	4.802917145	3.913951968	0.009923256
1-Palmitoyl-2-hydroxy-sn-glycero-3-phosphoethanolamine	7.895709243	1.730506585	0.011504913
Linoleoyl ethanolamide	4.264261586	1.525606188	0.015549134
Apigenin	1.435246498	1.995427455	0.016745868
Taurodeoxycholic acid	1.557423329	0.369278839	0.01772532
Sphingomyelin (d18:1/18:0)	2.011577089	0.376845755	0.020599453
Arg-Phe	1.360760278	1.853909223	0.025631739
Cholecalciferol (Vitamin D3)	2.201414803	0.718335197	0.026329684
Ile-Asp	1.103575983	1.861085991	0.030442129
Phytosphingosine	1.503945999	0.633606652	0.044928183
Nicotinamide	4.556223721	2.299255042	0.047195005
L-Pipecolic acid	2.695729342	0.627382005	0.050111035
Kynurenic acid	1.892297247	1.547235382	0.05533722
Purine	2.275966976	3.804678508	0.060011685
1,2-dioleoyl-sn-glycero-3-phosphatidylcholine	2.631108561	0.318717333	0.060661309
Sphingosine	4.872316938	0.623011293	0.062018291
Adenine	8.92867391	0.435705963	0.062349759
1-Stearoyl-2-oleoyl-sn-glycerol 3-phosphocholine (SOPC)	1.091260417	0.607281917	0.065743681
N6-Methyladenine	2.57709008	0.649648323	0.068533071
Xanthine	2.427351279	1.999870423	0.071269264
Thymine	2.239259825	1.624038378	0.075919083
Met-Arg	1.236511509	0.55820721	0.082418829
Lys-Leu	2.121138813	1.782006193	0.086689458
Indole-2-carboxylic acid	13.08410638	0.203641073	0.092410124
