# Supplementary material for: 919 Syrup Alleviates Postpartum Depression by Modulating the Structure and Metabolism of Gut Microbes and Affecting the Function of the Hippocampal GABA/Glutamate System
Source: Front Cell Infect Microbiol. 2021 Aug 20;11:694443. doi: 10.3389/fcimb.2021.694443 (PMC8417790; doi:10.3389/fcimb.2021.694443)
Supplement: Supplementary file 7 [file DataSheet_3.pdf]

	PPD vs CON			919 TJ vs PPD			
	log2FoldChange	Fold change	p-value	log2FoldChange	Fold change	p-value	description
ENSMUSG00000010803	0.0618	1.043767221	0.30721	0.0444	1.031254204	0.38166	Gabra1
ENSMUSG00000000560	-0.0181	0.987532409	0.85954	0.1014	1.072814023	0.33869	Gabra2
ENSMUSG00000031343	0.1369	1.099539932	0.42404	-0.0978	0.934456881	0.49903	Gabra3
ENSMUSG00000029211	-0.0209	0.985617652	0.74564	0.0368	1.025835924	0.50345	Gabra4
ENSMUSG00000055078	-0.1728	0.887119277	0.26238	0.0721	1.051245773	0.63179	Gabra5
ENSMUSG00000020428	-2.4206	0.186778461	0.073779	0.0537	1.037923418	0.97595	Gabra6
ENSMUSG00000029212	0.0633	1.044853011	0.60048	-0.0433	0.970432653	0.74349	Gabrb1
ENSMUSG00000007653	0.0904	1.064665329	0.35511	-0.1356	0.910291175	0.14316	Gabrb2
ENSMUSG00000033676	0.0043	1.002984979	0.94777	-0.018	0.987600861	0.78666	Gabrb3
ENSMUSG00000001260	0.0956	1.068509696	0.23504	-0.1097	0.92678076	0.13623	Gabrg1
ENSMUSG00000020436	-0.0006	0.999584198	0.98762	0.0121	1.008422351	0.71914	Gabrg2
ENSMUSG00000055026	0.0774	1.055114816	0.315	-0.0573	0.961061061	0.46409	Gabrg3
ENSMUSG00000029054	0.0528	1.03727613	0.77645	-0.1202	0.920060094	0.49192	Gabrd
ENSMUSG00000031340	0.4426	1.359051381	0.26834	0.4313	1.348448104	0.20767	Gabre
ENSMUSG00000031344	-0.44	0.737134609	0.36111	-0.3004	0.812027223	0.44106	Gabrq
ENSMUSG00000020159	0.1476	1.107725179	0.96014	0.644	1.56265576	0.72136	Gabrp
ENSMUSG00000028280	-1.0249	0.491444362	0.058171	-0.2956	0.814733424	0.67089	Gabrr1
ENSMUSG00000023267	0.5637	1.478055046	0.0043494	-0.289	0.818469182	0.23125	Gabrr2
ENSMUSG00000074991	-0.3674	0.775178252	0.59673	0.7762	1.712613971	0.23382	Gabrr3
ENSMUSG00000024462	0.0593	1.041960076	0.0313	-0.0511	0.96520012	0.054383	Gabbr1
ENSMUSG00000039809	0.0447	1.031468669	0.28985	-0.0372	0.974544521	0.40222	Gabbr2
ENSMUSG00000020524	0.0125	1.008701984	0.92643	0.0195	1.013608129	0.88219	Gria1
ENSMUSG00000033981	0.0534	1.03770761	0.30403	-0.0289	0.980167353	0.59048	Gria2
ENSMUSG00000001986	0.0729	1.051828869	0.19924	-0.0364	0.975085073	0.49377	Gria3
ENSMUSG00000025892	0.1297	1.094066173	0.29927	-0.05	0.965936329	0.62858	Gria4
ENSMUSG00000041078	0.0337	1.02363402	0.54453	-0.0411	0.971913618	0.48019	Grid1
ENSMUSG00000071424	-0.0373	0.974476973	0.63036	-0.0677	0.954157941	0.39618	Grid2
ENSMUSG00000010825	0.1166	1.08417677	0.65088	-0.1065	0.928838707	0.60727	Grid2ip
ENSMUSG00000022935	-0.1071	0.928452495	0.40129	0.0312	1.021861733	0.76657	Grik1
ENSMUSG00000056073	0.008	1.00556058	0.92194	0.027	1.018891197	0.75607	Grik2
ENSMUSG00000001985	-0.0699	0.952704032	0.63043	-0.1396	0.907770808	0.25048	Grik3
ENSMUSG00000032017	0.0298	1.020870593	0.797	0.0291	1.020375384	0.80444	Grik4
ENSMUSG00000003378	-0.0397	0.972857226	0.15853	-0.0722	0.951186405	0.020331	Grik5
ENSMUSG00000022564	0.0481	1.033902398	0.38443	-0.0424	0.971038229	0.38179	Grina
ENSMUSG00000026959	0.0411	1.028898023	0.28446	-0.0742	0.949868694	0.040437	Grin1
ENSMUSG00000085830	0.0075	1.00521214	0.94668	-0.0411	0.971913618	0.66874	Grin1os
ENSMUSG00000059003	0.084	1.059952783	0.52243	-0.1203	0.919996323	0.33946	Grin2a
ENSMUSG00000030209	0.0979	1.070214514	0.38693	-0.1732	0.88687335	0.15108	Grin2b
ENSMUSG00000020734	-0.0869	0.941543725	0.21376	0.0364	1.025551542	0.57389	Grin2c
ENSMUSG00000002771	-0.1177	0.921655819	0.21964	0.0701	1.049789447	0.51164	Grin2d
ENSMUSG00000039579	-0.1142	0.923894485	0.41609	-0.0104	0.99281719	0.92214	Grin3a
ENSMUSG00000035745	0.1445	1.105347503	0.71034	-0.4058	0.754817617	0.33537	Grin3b
ENSMUSG00000034813	0.0948	1.067917353	0.19621	0.0209	1.014592218	0.76924	Grip1
ENSMUSG00000030098	0.1749	1.128886154	0.25239	-0.1009	0.932451117	0.50719	Grip2
ENSMUSG00000085418	-0.213	0.862741345	0.38166	0.0239	1.016704198	0.91483	Grip1os1
ENSMUSG00000084764	0.2684	1.204471285	0.84831	0.1543	1.112881514	0.88948	Grip1os2
ENSMUSG00000085454	-0.2535	0.83885886	0.56075	-0.3288	0.796198468	0.47839	Grip1os3
ENSMUSG00000019828	0.0574	1.040588739	0.6049	0.012	1.008352455	0.89955	Grm1
ENSMUSG00000023192	-0.0481	0.967209285	0.64867	-0.0992	0.933550518	0.2365	Grm2
ENSMUSG00000003974	-0.1266	0.915987617	0.2494	-0.1588	0.895769842	0.18644	Grm3
ENSMUSG00000063239	-0.1315	0.912881814	0.39578	-0.0206	0.985822627	0.88136	Grm4
ENSMUSG00000049583	0.0671	1.047608739	0.43738	0.0164	1.011432471	0.86184	Grm5
ENSMUSG00000000617	-0.0289	0.980167353	0.97979	0.3063	1.236532364	0.80572	Grm6
ENSMUSG00000056755	-0.0296	0.979691888	0.6784	0.0323	1.022641161	0.62821	Grm7
ENSMUSG00000024211	0.048	1.033830736	0.7983	0.1088	1.078330933	0.47085	Grm8
ENSMUSG00000024935	7.00E-04	1.000485321	0.98545	0.0067	1.004654887	0.87673	Slc1a1

ENSMUSG00000005089	0.0453	1.031897734	0.55743	-0.1068	0.928645581	0.091508	Slc1a2
ENSMUSG00000005360	-0.0511	0.96520012	0.27551	-0.0318	0.978199071	0.34072	Slc1a3
ENSMUSG00000020142	0.013	1.009051634	0.76771	0.0191	1.013327136	0.66469	Slc1a4
ENSMUSG00000001918	-0.2105	0.864237657	0.21037	0.2116	1.157971704	0.21628	Slc1a5
ENSMUSG00000005357	0.0714	1.050735829	0.55132	0.0705	1.050080551	0.46563	Slc1a6
ENSMUSG00000030310	0.0543	1.038355168	0.35454	-0.0235	0.983842989	0.61496	Slc6a1
ENSMUSG00000055368				-1.0941	0.468428253	0.4253	Slc6a2
ENSMUSG00000021609	-0.1945	0.873875706	0.70577	0.1476	1.107725179	0.72781	Slc6a3
ENSMUSG00000020838	0.7245	1.652327884	0.30424	0.2765	1.211252799	0.69253	Slc6a4
ENSMUSG00000030307	-0.1085	0.927551956	0.12002	-0.0311	0.978673812	0.5833	Slc6a11
ENSMUSG00000030109	0.7676	1.702435335	0.05266	0.2634	1.200304131	0.59445	Slc6a12
ENSMUSG00000030108	0.4917	1.406100779	0.17849	0.1119	1.080650494	0.80814	Slc6a13
ENSMUSG00000008932	-0.3415	0.789220316	0.6398	0.4318	1.348915522	0.53375	Slc1a7
ENSMUSG00000019082	-0.0116	0.991991731	0.84303	-0.0018	0.998753113	0.97609	Slc25a22
ENSMUSG00000021185	0.0407	1.028612792	0.66367	-0.0731	0.950593209	0.39528	Dglucy
ENSMUSG00000070880	0.1044	1.075047198	0.050038	-0.0046	0.996816601	0.91927	Gad1
ENSMUSG00000087264	0.0645	1.045722457	0.89908	-0.1405	0.907204688	0.77659	Gad1os
ENSMUSG00000090665	0.1456	1.106190609	0.71427	-0.5204	0.697178507	0.19702	Gad1-ps
ENSMUSG00000056880	-0.3361	0.792179899	0.48493	-0.407	0.754190038	0.43406	Gadl1
ENSMUSG00000026787	0.1512	1.11049277	0.009194	0.0098	1.006815966	0.87449	Gad2
ENSMUSG00000032350	-9.00E-04	0.999376362	0.97609	0.0031	1.002151066	0.9188	Gclc
ENSMUSG00000028124	0.0283	1.019809724	0.50563	0.0442	1.031111251	0.17825	Gclm
ENSMUSG00000026473	0.125	1.090507733	0.0038272	-0.01	0.993092495	0.78333	Glul
ENSMUSG00000021794	-0.0199	0.986301067	0.58753	-0.0327	0.977589029	0.26564	Glud1
ENSMUSG00000108799	0.1603	1.117519496	0.38554	-0.1359	0.910101905	0.47074	Glud-ps
ENSMUSG00000048217	-0.171	0.888226796	0.71275	-0.2422	0.845455074	0.5915	Nags
ENSMUSG00000040459	-0.0257	0.982343847	0.6279	0.0965	1.069176475	0.028878	Arglu1
ENSMUSG00000051978	0.0394	1.027686335	0.73028	0.1396	1.101599645	0.20519	Erich1
ENSMUSG00000075302	4.00E-04	1.000277297	0.99893	-0.2781	0.824676384	0.3325	Erich2
ENSMUSG00000086515	-0.1938	0.874299816	0.7761	0.3318	1.258582685	0.6318	Erich2os
ENSMUSG00000078161	-0.0849	0.942849887	0.22553	0.0972	1.069695369	0.12739	Erich3
ENSMUSG00000074261	-0.2225	0.857078944	0.82057	-0.3232	0.799295017	0.74832	Erich4
ENSMUSG00000044726	0.2502	1.189371986	0.40478	-0.138	0.908778116	0.62214	Erich5
ENSMUSG00000070471	-0.0539	0.963328665	0.67344	-0.0368	0.97481476	0.74812	Erich6
ENSMUSG00000022002	-2.2132	0.215655438	0.19637	2.1031	4.296315674	0.15163	Erich6b
ENSMUSG00000074579	0.0994	1.071327817	0.31126	0.0664	1.04710056	0.43831	Lekr1
ENSMUSG00000063163				0.5987	1.514351383	0.84376	Speer2
ENSMUSG00000067855	0.1796	1.132569827	0.95285	-0.331	0.794985251	0.91322	Speer3
ENSMUSG00000073119	-0.915	0.530343871	0.11206	0.0948	1.067917353	0.88068	Speer4a
ENSMUSG00000048703	-0.2991	0.812759264	0.3117	-0.0466	0.968215436	0.881	Speer4b
ENSMUSG00000073208	-0.4295	0.742519078	0.88755				Speer4c
ENSMUSG00000089871				0.2781	1.212596867	0.92706	Speer4cos
ENSMUSG00000070933	-0.7501	0.594562344	0.80495				Speer4d
ENSMUSG00000091255	-0.4295	0.742519078	0.88755	0.2781	1.212596867	0.92706	Speer4e
ENSMUSG00000058643				0.2781	1.212596867	0.92706	Speer4f1
ENSMUSG00000091358	-0.2445	0.844108291	0.84493	0.8855	1.847404772	0.42738	Speer5-ps1
ENSMUSG00000091304				0.2781	1.212596867	0.92706	Speer6-ps1
ENSMUSG00000107956	-1.9777	0.253894316	0.20094	1.6841	3.213398715	0.14619	Speer9-ps1
ENSMUSG00000047025	1.4114	2.659951609	0.26015	-0.118	0.921464186	0.92703	Ccer1
ENSMUSG00000096257	-0.6445	0.63971446	0.10558	0.0108	1.00751408	0.97851	Ccer2
ENSMUSG00000053801	-0.0704	0.952373907	0.33546	-0.0143	0.990136957	0.84484	Grwd1
ENSMUSG00000030680	0.1155	1.083350441	0.94838	-0.5875	0.665495124	0.79578	Pagr1a
ENSMUSG00000092534	1.4792	2.787940941	0.2603	-0.3462	0.786653384	0.7456	Pagr1b
